# Colonoscopy in Amoebic Liver Abscess: Clinical Significance of Colonic Pathology

**DOI:** 10.7759/cureus.78632

**Published:** 2025-02-06

**Authors:** Mohmad Sejarali Sayeed, Swapnil Saikhedkar, Jay Prakash S Rajput, Pooja Parmar, Sajidali S Saiyad, Tehsin Saiyad, Chetan Kumar R, Nehal Shah

**Affiliations:** 1 Department of Gastrointestinal Surgery, Aryavart Hospital, Meerut, IND; 2 Department of Physiology, Pacific Medical College and Hospital, Udaipur, IND; 3 Department of Nursing, J and D Institute of Nursing, Surat, IND; 4 Department of Microbiology, Veer Narmad University, Surat, IND; 5 Department of Forensic Medicine, Rajmata Vijaya Raje Scindia (RVRS) Government Medical College, Bhilwara, IND; 6 Department of Pharmacology, Dr. M.K. Shah Medical College and Research Centre, Ahmedabad, IND

**Keywords:** amoebic liver abscess, colonic pathology, colonoscopic findings, entamoeba histolytica, extraintestinal amoebiasis

## Abstract

Introduction

Amoebiasis, caused by *Entamoeba histolytica*, remains a significant global health concern, particularly in endemic regions such as India. An amoebic liver abscess (ALA) is the most common extraintestinal manifestation of amoebiasis, often linked to colonic pathology. However, the correlation between hepatic and colonic involvement is underexplored, leading to missed diagnostic opportunities and suboptimal management. This study investigates the clinical significance of colonic pathology in ALA patients through colonoscopic evaluations.

Materials and methods

This observational study included 110 ALA patients evaluated over 3.5 years in a tertiary care hospital in Gujarat, India. Patients underwent colonoscopy within 48 hours of initiating anti-amoebic treatment. Data on demographic characteristics, clinical presentations, and colonoscopic findings were analyzed using descriptive and inferential statistical methods. Inclusion criteria encompassed adult patients diagnosed with ALA, while those with pyogenic liver abscesses or contraindications to colonoscopy were excluded. The ethical approval and informed consent ensured compliance with research standards.

Results and discussion

Colonic abnormalities were identified in 55.45% of patients with ALA, with ulceration being the most common (33.64%). Larger abscesses (>10 cm) and multiple liver abscesses were significantly associated with colonic pathology (p = 0.002 and p = 0.019, respectively), particularly in the right lobe (p = 0.037). Middle-aged patients (31-50 years) had the highest prevalence (100%), while males were more affected than females (4.64:1), likely due to risk factors like alcoholism and obesity. Patients with multiple abscesses had greater odds of colonic findings (OR = 3.09), as did those with larger abscesses (OR = 4.67). These findings emphasize the importance of colonoscopy in high-risk patients to improve diagnosis and treatment strategies.

Conclusion

This study links colonic pathology in ALA to age, sex, and abscess characteristics, highlighting higher prevalence in middle-aged males and those with larger or multiple abscesses. Despite limitations, it underscores colonoscopy’s role in improving diagnosis and management.

## Introduction

Amoebiasis, caused by *Entamoeba histolytica*, is a major health problem worldwide, with approximately 50 million people infected and 40,000 to 100,000 deaths reported annually [1]. In India, where amoebiasis is considered endemic, the rate of infection due to *E. histolytica* was within the range of 11.7% to 18.5% [2]. These alarming figures indicate the high prevalence rates for this parasitic infection, especially in places with inadequate clean water facilities and health sanitation.

Amoebiasis, though preventable and curable, remains one of the major challenges to healthcare systems, especially in low- and middle-income countries [3]. Amoebiasis has been historically recognized since the late 19th century, and its causative organism, *E. histolytica*, was identified in 1875 by Friedrich Lösch. Over the ensuing decades, its spectrum of clinical manifestations has been documented from asymptomatic colonization to invasive diseases such as amoebic liver abscess (ALA) [4,5].

The most common extraintestinal complication of amoebiasis is ALA, usually due to the hematogenous spread of trophozoites from the colon to the liver via the portal vein. However, involvement of the colon in ALA patients may remain asymptomatic [6] or may be presented as ulcers, inflammation, or even dysplasia and is underreported and probably underdiagnosed [7,8]. Available evidence suggests that colonic lesions in ALA patients have significant clinical importance, whose incidences are reported to be as high as 58% to 77% by various works [7,8]. It is, therefore, very puzzling that there is a limited understanding of the relationship between the hepatic and colonic manifestations of amoebiasis.

Colonoscopic examination not only helps in the diagnosis of amoebic colitis but also differentiates it from other gastrointestinal pathologies, thus helping to intervene appropriately and on time to prevent morbidity [9]. This knowledge gap impacts the clinician's ability to provide holistic care since failure to identify colonic lesions may result in complications such as persistent infection, misdiagnosis, and therapeutic failure. This study addresses the important need to investigate colonic involvement in patients with ALA. The findings could have an important impact on clinical practice by improving diagnostic protocols and treatment strategies. Better diagnosis and management of colonic lesions in ALA patients may not only decrease morbidity but also provide insights into the broader pathogenesis of amoebiasis.

The study will carry public health importance in endemic regions such as India, where better comprehension of this relationship might help formulate targeted interventions and resource allocation. This study tries to fill the knowledge gap to further the wheel of clinical practice and join the global effort in reducing the burden of amoebiasis.

## Materials and methods

The present study was undertaken to present the colonoscopic findings in 110 patients diagnosed with ALA over 3.5 years, starting from January 2017 to August 2020, at a tertiary care hospital in Surat, Gujarat, India. The study utilized both retrospective and prospective observational analyses as part of its design. In this context, retrospective data were retrieved from the hospital records, while prospective data were derived from patients enrolled during the period of the study.

The study population consisted of adult patients aged 18 years or more who were being treated for liver abscess amoebiasis. Inclusion criteria require persons aged 18 years and above with a confirmed diagnosis of ALA. Such exclusion criteria include all those patients having pyogenic liver abscesses, those being unfit or even contraindicated for undergoing colonoscopy, and those women who become pregnant.

The ethical considerations have been taken with due care. The approval was taken from Unique Hospital-Multispeciality and Research Institute, Surat (Approval Number: IEC/APPROVAL/20160107; dated: 27/09/2016). Informed consent was obtained in the prospective arm while the retrospective arm was conducted under an ethics committee-approved waiver of consent.

Data collection involved a total of 110 patients, of whom 49 were retrospective, while 61 were prospective. Demographic data, clinical presentation, laboratory, microbiological, radiological, and colonoscopic findings for the retrospective cases were collected from their medical records, while it was directly obtained from patients in the case of prospective ones after their informed consent.

The diagnosis of ALA was mainly made from a combination of clinical presentation with the aid of supporting laboratory investigations, including hematological, biochemical, and microbiological tests, along with radiological imaging such as ultrasonography or computed tomography scans. All patients received anti-amoebic treatment as per the standard protocol along with symptomatic and supportive management. A colonoscopy was carried out within 48 hours after the start of anti-amoebic therapy when bowel preparation was adequately done, and histopathological reports on biopsy specimens were documented.

Outcome measurement was followed up until discharge, including age, sex, occupation, clinical presentations, laboratory parameters like complete blood count and liver function tests, colonoscopic and histopathological findings, associated co-morbidities, morbidity, mortality, recovery period, and duration of hospital stay.

The sample size for the study was determined based on the estimation of proportions. A prevalence rate of 58.82% for colonoscopic findings in ALA patients, as reported by Sachdev et al. [7], was used. The calculation assumed a 95% confidence level and a margin of error of ±10%. Based on these parameters, the required sample size was estimated to be approximately 93 participants. To account for potential dropouts or missing data, the calculated sample size of 93 was increased by 15%, resulting in a final target sample size of 110 patients.

Descriptive statistical analysis was done using SPSS version 24 (IBM Corp., Armonk, NY), while graphical representation was prepared in Microsoft Excel (Microsoft Corporation, Redmond, WA). Qualitative data are reported as frequencies and percentages. Quantitative data are expressed as mean with standard deviation or median.

## Results

In this study, 110 patients diagnosed with ALA were evaluated for colonic involvement through colonoscopy. The mean age of the patients was 48.2 ± 13.18 years, ranging from 18 to over 60 years. Colonoscopic findings were observed in 62 patients (56.4%), while 48 patients (43.6%) had unremarkable findings. Among patients aged 18-30 years (40% of the total, n = 44), colonic findings were present in 18 patients (41%) and absent in 26 patients (59%). In the 31-40 years age group (10%, n = 11), all patients had colonic involvement (100%, n = 11). Similarly, in the 41-50 years group (16%, n = 18), colonic pathology was found in all patients (100%, n = 18). For patients aged 51-60 years (20%, n = 22), colonic findings were evenly distributed, with 11 patients (50%) showing involvement and 11 patients (50%) showing no involvement. In the oldest group, those over 60 years (14%, n = 15), colonic findings were observed in four patients (27%), while 11 patients (73%) had no findings.

Among 110 patients, the clinical presentation of the disease varied, reflecting systemic effects. Abdominal pain occurred in 94.55% (n = 104), fever in 93.64% (n = 103), anorexia in 46.36% (n = 51), nausea in 40.0% (n = 44), diarrhea in 30.0% (n = 33), pallor in 29.09% (n = 32), jaundice in 20.91% (n = 23), and rales in 18.18% (n = 20). Among female patients (23.6%, n = 26), 11 (42.3%) had colonic findings, while 15 (57.7%) had no pathology. Among male patients (76.4%, n = 84), 51 (60.7%) showed colonic pathology, while 33 (39.3%) showed no abnormalities.

**Figure 1:**
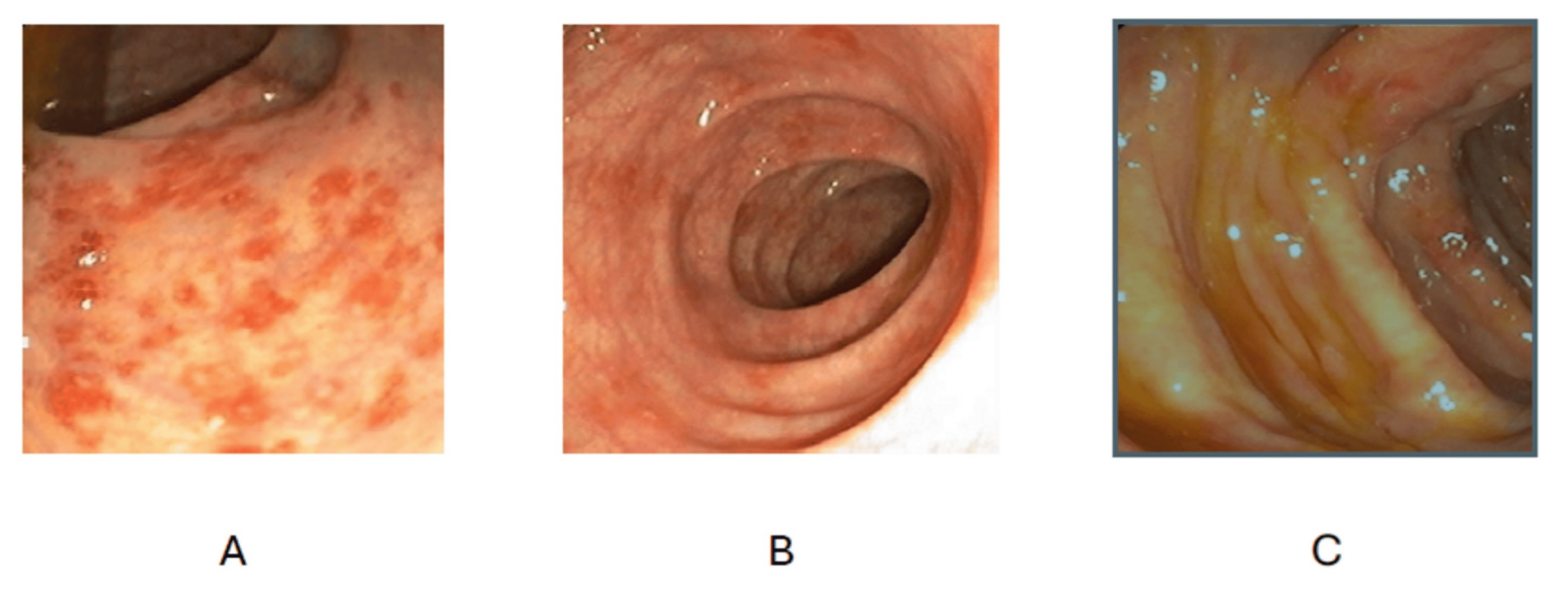
Endoscopic images demonstrating (A) an ascending colon ulcer showing characteristic erythematous and ulcerated areas, (B) edematous mucosa in the colon, and (C) inflammation in the ascending colon.

The study found that 49 patients (44.55%) had normal colonoscopic findings, while 61 patients (55.45%) exhibited abnormal findings. Among abnormal findings, ulceration was the most common, observed in 33.64% (n = 37), followed by edematous mucosal lesions and inflammation in 6.36% each (n = 7 each). Other abnormalities, including hemorrhoids and fissures, were noted in 9.09% (n = 10) (Figure 1).

**Figure 2:**
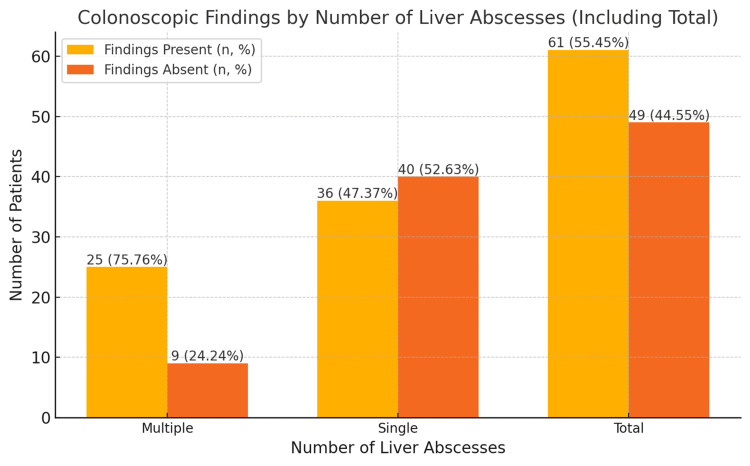
Relationship between liver abscess types (multiple, single, and total) and the presence or absence of colonoscopic findings, expressed as percentages. 1. The data in the bar chart are represented as N (count) and percentage (%). 2. The figure illustrates the distribution of colonoscopic findings (present and absent) by the number of liver abscesses (multiple, single, and total). 3. Statistical analysis was conducted using the chi-square test (χ2) to evaluate the association between the number of liver abscesses and colonoscopic findings. 4. P-values < 0.05 are considered statistically significant.

As shown in Table 1 and Figure 2, among 110 analyzed patients, 34 (30.91%) had multiple liver abscesses, and 76 (69.09%) had single abscesses. In patients with multiple abscesses, colonoscopic findings were observed in 25 (75.76%), and in patients with single abscesses, findings were present in 36 (47.37%). Conversely, nine (24.24%) patients with multiple abscesses and 40 (52.63%) with single abscesses had no colonoscopic findings. The chi-square test showed a significant association between the type of liver abscess and colonoscopic findings (χ² = 5.49, p = 0.019).

**Table 1:** Distribution of colonoscopic findings in patients with liver abscesses. 1. Data are represented as N (count) and percentage (%). 2. Statistical analysis was conducted using the chi-square (χ2) test. 3. P-values < 0.05 are considered statistically significant. 4. Test statistics (χ2) are presented in the second-to-last column.

Number of liver abscesses	Colonoscopic findings present (n, %)	Colonoscopic findings absent (n, %)	Total (n, %)	Statistic (χ2)	p-value
Multiple	25 (75.76%)	9 (24.24%)	34 (100%)	5.49	0.019
Single	36 (47.37%)	40 (52.63%)	76 (100%)		
Total	61 (55.45%)	49 (44.55%)	110 (100%)		

**Figure 3:**
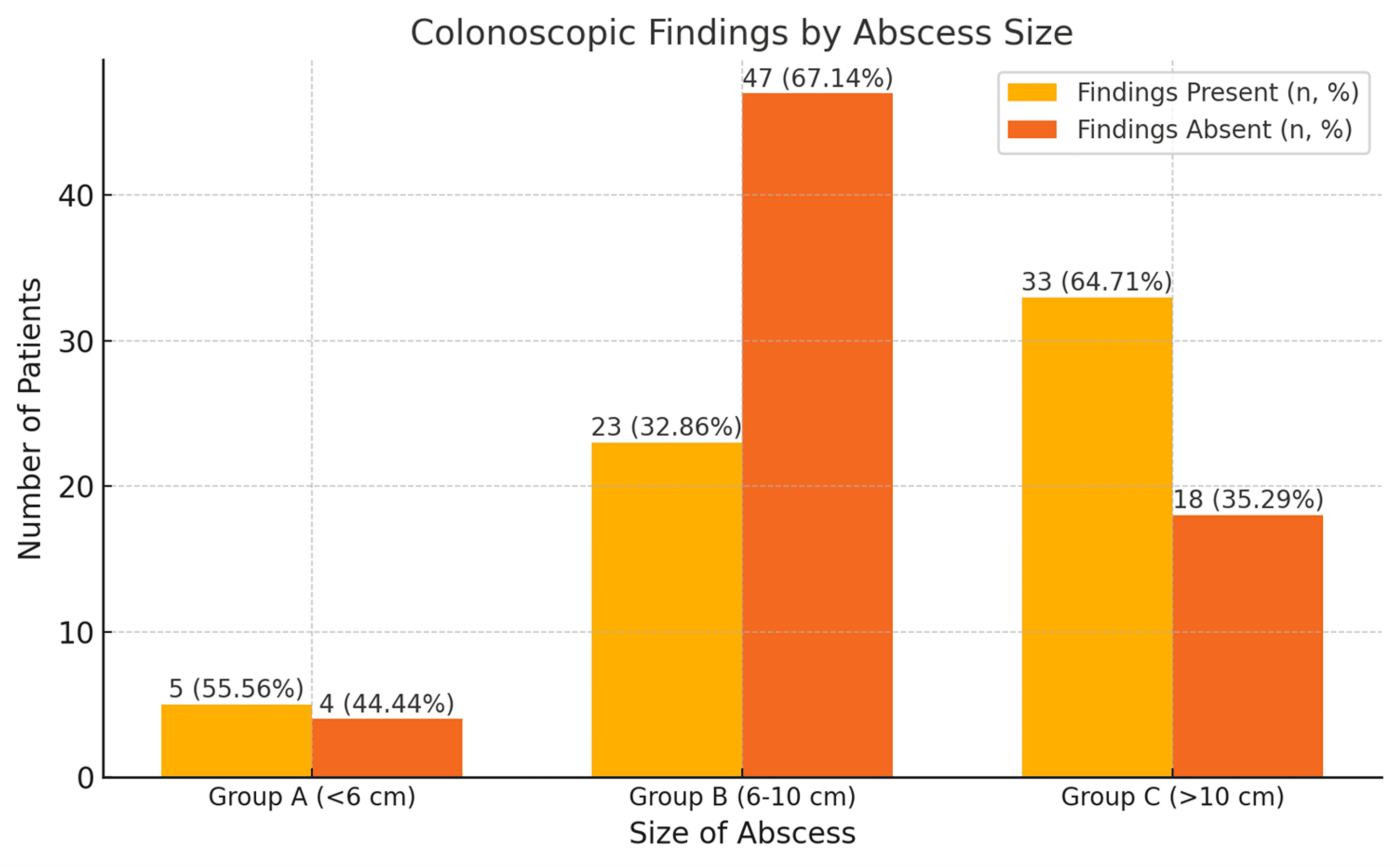
Relationship between the size of abscess and the percentage of colonoscopic findings present or absent. 1. The data in the bar chart are represented as N (count) and percentage (%). 2. The figure illustrates the distribution of colonoscopic findings (present and absent) by abscess size (Group A: <6 cm; Group B: 6–10 cm; Group C: >10 cm). 3. Statistical analysis was conducted using the chi-square test (χ2) to evaluate the association between abscess size and colonoscopic findings. 4. P-values < 0.05 are considered statistically significant.

The analysis also revealed a significant association between abscess size and colonoscopic findings (χ² = 12.31, p = 0.002) (Table 2 and Figure 3). Stratified by abscess size, findings were observed in 55.56% (n = 5) of group A (<6 cm), 32.86% (n = 23) of group B (6-10 cm), and 64.71% (n = 33) of group C (>10 cm) patients. Conversely, findings were absent in 44.44% (n = 4) of group A, 67.14% (n = 47) of group B, and 35.29% (n = 18) of group C patients. Larger abscesses were significantly more likely to show findings, with group C being 2.55 times more likely than group A and 3.74 times more likely than group B.

**Table 2:** Abscess size and associated colonoscopic observations. 1. Data are represented as N (count) and percentage (%). 2. Statistical analysis was conducted using the chi-square test (χ2) to calculate the p-values and assess the association between the affected lobe and colonoscopic findings. 3. P-values < 0.05 are considered statistically significant. 4. Test statistics (χ2) are presented in the second-to-last column.

Size of abscess	Colonoscopic findings present (n, %)	Colonoscopic findings absent (n, %)	Total (n, %)	Test statistic (χ2)	p-value
Group A (<6 cm)	5 (55.56%)	4 (44.44%)	9 (100%)		
Group B (6-10 cm)	23 (32.86%)	47 (67.14%)	70 (100%)		
Group C (>10 cm)	33 (64.71%)	18 (35.29%)	51 (100%)		
Total	61 (55.45%)	49 (44.55%)	110 (100%)	12.31	0.002

**Figure 4:**
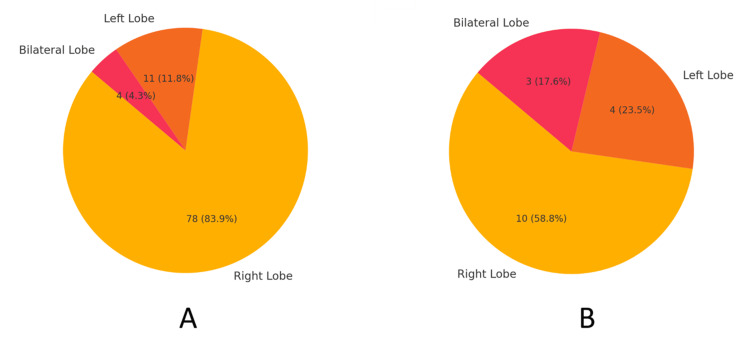
Involved lobe: (A) colonoscopic findings present; (B) colonoscopic findings absent. 1. The data in the pie charts are represented as N (count) and percentage (%). 2. The figures illustrate the distribution of affected lobes (right lobe, left lobe, and bilateral lobe) for colonoscopic findings. A. Left chart: colonoscopic findings present. B. Right chart: colonoscopic findings absent. 3. Statistical analysis was conducted using the chi-square test (χ2) to evaluate the association between the affected lobe and colonoscopic findings. 4. P-values < 0.05 are considered statistically significant. Test statistic: χ2 = 6.59, p = 0.0370, indicating a significant association at p < 0.05.

Of the 110 patients, abscess location was distributed as follows: right lobe (n = 88, 88.64%), left lobe (n = 15, 13.64%), and bilateral lobes (n = 7, 6.36%). Colonoscopic findings were present in 54.55% (n = 48) of patients with right lobe abscesses, 73.33% (n = 11) with left lobe abscesses, and 57.14% (n = 4) with bilateral lobe abscesses. Findings were absent in 45.45% (n = 40) of patients with right lobe abscesses, 26.67% (n = 4) with left lobe abscesses, and 42.86% (n = 3) with bilateral lobe abscesses (Table 3 and Figure 4). There was a statistically significant association between the lobe involved and colonoscopic findings (p = 0.037, χ² = 6.593, df = 2).

**Table 3:** Colonoscopic findings based on the affected lobe of the liver. 1. Data are represented as N (count) and percentage (%). 2. Statistical analysis was conducted using the chi-square test (χ2) to evaluate the association between the affected lobe and colonoscopic findings. 3. P-values < 0.05 are considered statistically significant. 4. The chi-square (χ2) test statistic is presented in the second-to-last column, and the corresponding p-value is provided in the last column.

Affected lobe	Colonoscopic findings present (n, %)	Colonoscopic findings absent (n, %)	Total (n, %)	Test statistic (χ2)	p-value
Right lobe	78 (88.64%)	10 (11.36%)	88 (100%)		
Left lobe	11 (73.33%)	4 (26.67%)	15 (100%)		
Bilateral lobe	4 (57.14%)	3 (42.86%)	7 (100%)		
Total	93 (84.55%)	17 (15.45%)	110 (100%)	6.59	0.0370

## Discussion

The study highlights remarkable age-based trends in colonic involvement among patients with ALA. The highest rates of colonic pathology occurred in middle-aged subjects aged 31-50 years. These results indicate a good correlation between age and colonic pathology in middle-aged subjects and signify that one must be alert for this group of patients [10]. In the 31-40 years age group (n = 11, 10% of total), all patients (100%, n = 11) exhibited colonic findings. Similarly, in the 41-50 years age group (n = 18, 16%), colonic pathology was observed in all patients (100%, n = 18). Among younger patients aged 18-30 years (n = 44, 40%), only 41% (n = 18) had colonic pathology, while the remaining 59% (n = 26) showed no findings. Patients over 60 years (n = 15, 14%) had the lowest rates, with colonic pathology detected in 27% (n = 4) and absent in 73% (n = 11). The even distribution in the 51-60 years age group (n = 22, 20%), i.e., 50% (n = 11) with findings and 50% (n = 11) without, suggests a transitional trend influenced by factors such as comorbidities or disease stage. Extensive colonic involvement in the younger [11] and middle-aged groups agrees with the active phase of the disease process and again underlines the importance of colonoscopy as a diagnostic modality.

Colonic pathology showed a significant male-to-female ratio of 4.64:1. Among males (n = 84, 76.4%), 60.7% (n = 51) had colonic findings, compared to 42.3% (n = 11) of females (n = 26, 23.6%). These differences, attributed to chronic alcoholism, diabetes, obesity, occupational exposure, and hormonal factors, align with findings by Katzenstein et al. [12] (25% of males with ALA were chronic alcoholics). These findings also align closely with the case series reported by Fleming et al., where 67% of the male patients had abnormal findings on colonoscopy, highlighting a similar pattern of male predominance in colonic involvement​ [13]. Yadav et al. delivered crucial insights about the elements of chronic alcoholism alongside diabetes and obesity and occupational hazards that affect ALA occurrence in men [14]. Research findings match our study data showing that males presented increased colonic pathologies while sharing a higher risk factor burden when compared to females. The strong consistency of research findings validates overall knowledge about how gender affects disease manifestation and demonstrates that behavioral and hormonal elements function as major determinants. The lower prevalence in females highlights the importance of thorough evaluations to prevent underdiagnosis.

Given the lower likelihood of colonic pathology in women, it becomes important that inpatient assessment be thorough, as a failure to do this due to implication of the symptoms may result in underdiagnosis of the female sex. This evidence is useful for building a detailed picture of sex-based differences in disease presentation and emphasizes the need to conduct further studies to explain the determinants behind these disparities.

Patients with multiple liver abscesses (n = 34, 30.91%) demonstrated a higher prevalence of colonoscopic abnormalities (75.76%, n = 25) compared to those with single abscesses (47.37%, n = 36). An odds ratio of 3.09 underscores the significant association between the number of abscesses and the likelihood of colonic findings, a pattern consistently observed in related research studies [8,15]. Larger abscesses (>10 cm, n = 51, 46.36%) were associated with a higher prevalence of positive colonoscopic findings (64.71%, n = 33) compared to smaller abscesses (<6 cm, n = 9, 8.18%), which showed findings in 55.56% (n = 5), a trend similarly reported by other researchers [16].

Right lobe abscesses (n = 88, 80%) were most associated with colonoscopic findings (54.55%, n = 48), compared to left lobe (73.33%, n = 11 of 15) and bilateral lobes (57.14%, n = 4 of 7). The statistical significance (p = 0.037) underscores regional predispositions linked to vascular anatomy and proximity to the cecum and ascending colon. The right lobe of the liver is more affected due to its direct vascular connection with the superior mesenteric vein, which drains the right side of the colon, and its larger size and metabolic activity, making it more susceptible to portal-venous-derived infections and inflammatory processes. Additionally, its proximity to the cecum and ascending colon enhances the likelihood of detecting associated pathologies via colonoscopy.

Qu et al. [17] reported a strong association between pyogenic liver abscesses and nonmetastatic colorectal cancers, particularly in Eastern Asia, highlighting the role of impaired colonic mucosal integrity in the hematogenous spread of bacteria to the liver. Their study recommends routine colonoscopic evaluation for patients with cryptogenic or unexplained liver abscesses to identify underlying colonic malignancies. Tovar et al. [18] highlighted the challenges in distinguishing colonic pathology from metastatic malignancies, underscoring the necessity of precise and detailed colonoscopic evaluation for accurate diagnosis and management, as also concluded by Prasad et al. (2022) [19]. However, the retrospective nature of this study, combined with its sample size limitations, calls for larger, prospectively designed studies to confirm these associations and explore the underlying mechanisms driving these patterns.

This study has several limitations that need to be addressed. Firstly, the retrospective nature of the analysis limits the ability to establish causal relationships between the observed variables and outcomes. Secondly, the sample size of 110 patients, although sufficient for preliminary insights, may not be large enough to generalize findings across diverse populations or detect less common patterns. Thirdly, the study predominantly relies on colonoscopic findings without considering other diagnostic modalities that could complement or refine the evaluation of colonic pathology, which could not be achieved in this study due to limited resources. Additionally, factors such as comorbidities, dietary habits, and socioeconomic conditions, which might influence the prevalence and severity of colonic involvement, were not fully explored. Another limitation is the potential for selection bias, as patients undergoing colonoscopy may not represent the broader population of individuals with ALA. Finally, while the study identifies significant associations, the lack of prospective data prevents robust conclusions about the progression and clinical significance of these findings. These limitations underscore the need for larger, multicentric, and prospectively designed studies to validate the results and investigate the underlying mechanisms driving the observed associations.

## Conclusions

The present study sheds light on the significant associations between colonic pathology and ALA, focusing on patient-related factors like age, sex, and clinical markers such as lobe involvement and disease severity. By combining both retrospective and prospective approaches, the research offers comprehensive insights into colonic abnormalities, emphasizing the pivotal role of colonoscopy in diagnosing these issues, particularly in middle-aged and male patients. The study also highlights the importance of disease severity indicators, like the number and size of liver abscesses, in identifying key risk factors. This dual-method approach aims to enhance diagnostic and management strategies for ALA.

However, the study has some limitations. The combined retrospective and prospective design, while thorough, may introduce variability in data collection and standardization. Additionally, the lack of biopsy results limits the ability to provide detailed insights into the underlying pathological changes, making it difficult to differentiate between various etiologies of colonic pathology. The relatively small sample size and absence of significant neoplastic findings further restrict the generalizability of the results. Future research should involve larger cohorts, the inclusion of biopsy data, and a focus on a broader range of pathological findings to confirm these associations and better understand the mechanisms at play. Despite these limitations, the study lays a solid foundation for further investigation and reinforces the importance of colonoscopy in the diagnosis and treatment of ALA.
